# Revision with Locking Compression Plate by Compression Technique for Diaphyseal Nonunions of the Femur and the Tibia: A Retrospective Study of 54 Cases

**DOI:** 10.1155/2021/9905067

**Published:** 2021-07-14

**Authors:** Peng Ding, Qiyu Chen, Changqing Zhang, Chen Yao

**Affiliations:** Department of Orthopaedics, Shanghai Jiaotong University Affiliated Shanghai Sixth People's Hospital, Shanghai 200233, China

## Abstract

Nonunion after diaphyseal fracture of the femur or the tibia is a common but difficult complication for treatment. Currently, the main treatment modalities include nail dynamization, exchange nailing, and bone transport, but revision with compression plating in these nonunions was rarely reported. To evaluate the outcomes of compression plating in the treatment of femur and tibia shaft nonunions, we retrospectively reviewed 54 patients with diaphyseal nonunion of the tibia or the femur treated with locking compression plate (LCP) by compression technique. There were 46 aseptic and 8 septic nonunions in the case series. Patient's history, fracture characteristics, previous interventions, and types of nonunion were recorded. The possible reason which might lead to nonunion was also analyzed for each case. Patients with aseptic nonunions were revised by hardware removal and compression plating with or without bone grafting. For septic nonunions, a two-stage surgery strategy was used. Compression plating with iliac crest bone grafting (ICBG) or free vascularized fibular grafting (FVFG) was used as the final treatment for septic nonunions. The compression technique and bone grafting method were individualized in each case according to the patient's history and architecture of the nonunion. Each patient finished at least a two-year follow-up, and all cases achieved healing uneventfully. Our study showed that compression plating with LCP was an effective method to treat diaphyseal nonunions of the tibia and the femur. It is compatible with different bone grafting methods for both infected and noninfected nonunions and is a good alternative to the current treatment methods for these nonunions.

## 1. Introduction

Long bone nonunions have a devastating impact on patient's quality of life and cause a high socioeconomic burden [1]. The occurrence of nonunion is multifactorial, and the mechanical and biological factors include inadequate immobilization, comminution, bone defects, poor vascularity of the fracture fragments, poor soft tissue envelop, and local infection [2]. Fracture personalities and patient backgrounds also play important roles in the development of nonunions [3]. Nowadays, with the rapid development of implants and surgical techniques, more fractures are being cured by surgeries when reliable stability and early mobilization are achieved. However, diaphyseal long bone nonunions of the tibia and the femur still remain a common complication [4] and are difficult to treat.

Currently, several techniques have been used to treat diaphyseal long bone nonunions including but not limited to nail dynamization, exchange nailing, augmentation plating, and bone transportation with external fixation [3]. Among these techniques, exchange nailing has been considered as a preferred choice with both biological and mechanical advantages. However, there were conflicting reports on its success [5] and its use also has limitations [6, 7]. Although compression plating has been described as a successful treatment for humeral shaft nonunions [8, 9], it is rarely mentioned in the treatment of tibia or femur diaphyseal nonunions [10]. Plating was even believed to produce poor results in treating these nonunions, especially in cases with infection and bone loss [3, 11]. In the current work, we retrospectively studied a series of tibia and femur diaphyseal nonunions revised by compression plating with locking compression plate. Our main purpose is to investigate the results of compression plating, emphasizing the essential techniques of compressing the bone gaps and selection of bone grafting methods in the treatment of nonunited femoral and tibial fractures. By highlighting the advantages of this technique, we also aim to explore compression plating as an alternative method for treating diaphyseal nonunions of the tibia and the femur.

## 2. Materials and Methods

This was a retrospective continuous study and was approved by the ethic committee of our hospital. All methods were carried out in accordance with guidelines of the institutional internal review board of our hospital. The following inclusion criteria were used: (1) tibia or femur nonunion at the area between the two diaphyseal-metaphyseal junction sites and treated by compression plating and (2) minimum of 2 years of radiological and clinical follow-up after treatment performed by our surgical team. Patients with congenital limb deformities, pathologic fractures, and nonunions following periprosthetic fractures were excluded. From 2011.1 to 2015.12, a total of 61 tibia and femur diaphyseal nonunion cases were treated by compression plating and 54 cases were enrolled in the study based on the above criteria. The excluded cases included one pathological femur fracture nonunion after multiple myeloma, one femur fracture with limb deformity after poliomyelitis, one case of periprosthetic femur fracture, and 4 cases which were lost to follow-up within 6 months after surgery.

The diagnosis of nonunion was based on clinical and radiological findings. Plain radiograph was the main method to identify nonunions. CT scan was done when nonunions were doubtful on X-rays. Generally, diaphyseal fractures failing to heal at 9 months with no progress during the previous 3 months were diagnosed as nonunion. We simply divided all nonunions into two categories: the nonseptic and the septic. These two categories will be discussed separately latter. The anatomical site involved included the area between the two diaphyseal-metaphyseal junction sites. The diaphyseal region was further divided into three parts: the proximal, middle, and distal third to better describe the location of nonunions. The background of patients, fracture details, and history of treatments were studied. AO/OTA classification was adopted to describe the pattern and severity of fractures. The Gustilo-Anderson (GA) classification was used to assess soft tissue damage for open fractures. The Weber and Cech classification was used to describe the morphology and biological conditions of the nonunions. X-rays after surgeries were studied to evaluate patients' initial fixations and fracture reductions. Problems which might lead to the development of nonunions were analyzed. Fracture gap that resulted from bone loss, comminution, or poor compression between fragments after plating or nailing was classified as “poor bony contact.” Bone defects which may need surgical reconstruction were also noted. Problems which might result in insufficient fracture stability, such as inappropriate choice of implants, undersized nail or plate, and insufficient screw purchase, were documented as “inappropriate fixation.” Local infections were diagnosed by radiologic study, elevated blood C-reactive protein (CRP) and erythrocyte sedimentation rate (ESR), and positive bacterial tests. We divided all infected nonunions into draining and nondraining types in order to define the status of infections.

### 2.1. Surgical Principles and Procedures

All the operations were planned and performed after careful evaluation of the presence of infection, associated bone loss, condition of the soft tissues, and stability of previous fixation. In our case series, compression plating was generally used to treat tibia and femur diaphyseal nonunions in the following conditions: (1) nonunion at the nonisthmus region or at the area with a significant widening of the medullary canal, (2) patient who failed revision by nailing, (3) nonunion previously fixed by an intramedullary nail which has the largest diameter as marketed by the manufacturer, and (4) nonunion with large bone defect which may require structural bone grafting. Poor soft tissue coverage was the main contraindication of plating.

**Figure 1:**
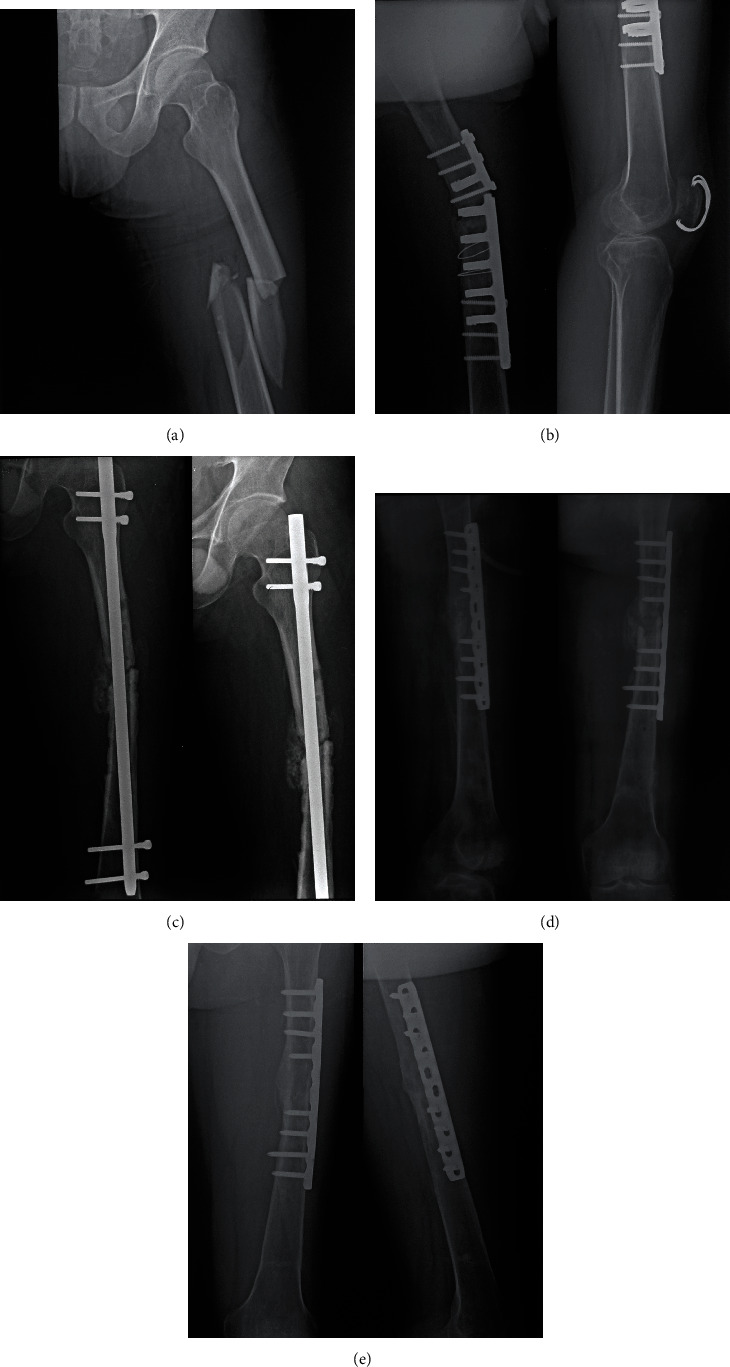
Case 35 in the aseptic group. (a) Radiograph showed the initial left femur shaft fracture. (b) Radiograph of the left femur 12 months after initial fixation showing breakage of implants (DCP) and nonunion. (c) X-rays showed the patient failed revision surgery with exchange nailing. Note the cortical defects and multiple screw holes left from previous surgeries. Prolonged nonunion and multiple surgical interventions may compromise bone quality and decrease purchase of regular cortical screws. (d) Immediate postoperative radiograph after revision surgery with compression plating and ICBG. Locking screws were used to increase purchase, and compression was performed using ATD in this case. (e) Radiograph of the left femur 8 months after revision showed healing of the fracture.

**Figure 2:**
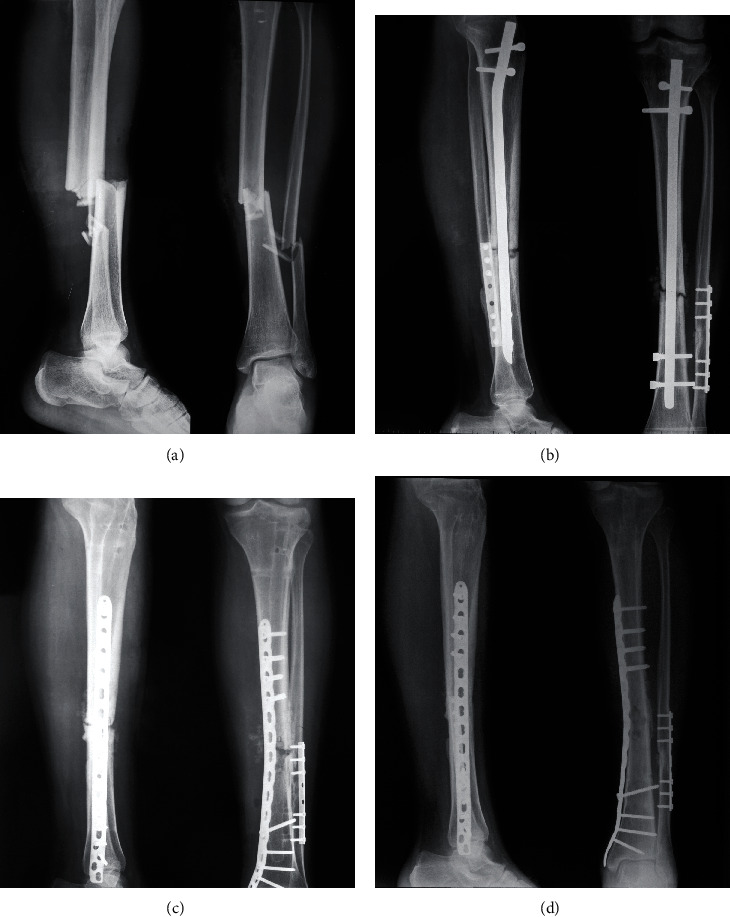
Case 20 in the aseptic group. (a) Radiograph showed initial left tibiofibular fracture. (b) Radiograph of the left tibia 13 months after initial fixation showed nonunion. Note the cortices were relatively intact on AP and lateral views. (c) Immediate postoperative radiograph after revision surgery. Compression by ATD combined with dynamic compression through LCP was applied to minimize the bone gap in this case. No bone grafting was used. (d) Radiograph of the left tibia and fibula 6 months after revision showed healing of the fracture.

All patients underwent surgical treatment with open reduction and internal fixation. With some minor variation in technique, the treatment of aseptic nonunion included removal of the previous fixation devices, excision of nonunions, correction of malalignments, and restabilization using locking compression plates (LCPs) with or without grafting (Figures 1 and 2). When there was no need of autogenous bone graft, we only refreshed the fragment edges with limited excision of nonunion and performed interfragmentary compression to procure healing. When iliac crest bone grafting (ICBG) was used, complete excision of the nonunion, freshening of the fragment edges, recanalization, and preparation of a healthy vascular bed for the grafting were performed.

For the nonunions with local infections, we adopted a two-stage surgery strategy. The first-stage operations included removal of internal fixation, debridement, restabilization with external fixator, and placement with antibiotic beads. Appropriate antibiotics were selected on the basis of the culture sensitivity reports and suggestions of infectious diseases specialists. The second-stage procedures were done after 6-8 weeks depending on the control of infection and local condition of the soft tissue. Compression plating with bone grafting was performed for the second surgery. Bioabsorbable bone grafting substitutes mixed with antibiotics were used intraoperatively to reduce the chance of recurrence of infection and to promote healing.

Application of plates followed the general principles of fracture management. The plate was put on the lateral aspect of the femur or the medial surface of the tibia. Generally, a ten-hole or longer LCP (Synthes) or Distal Femur-LCP (DF-LCP, Synthes) was chosen for each case. Appropriate contouring was performed to fit plates to the bone surfaces if necessary. Any malalignment was corrected before completing the final fixation.

**Figure 3:**
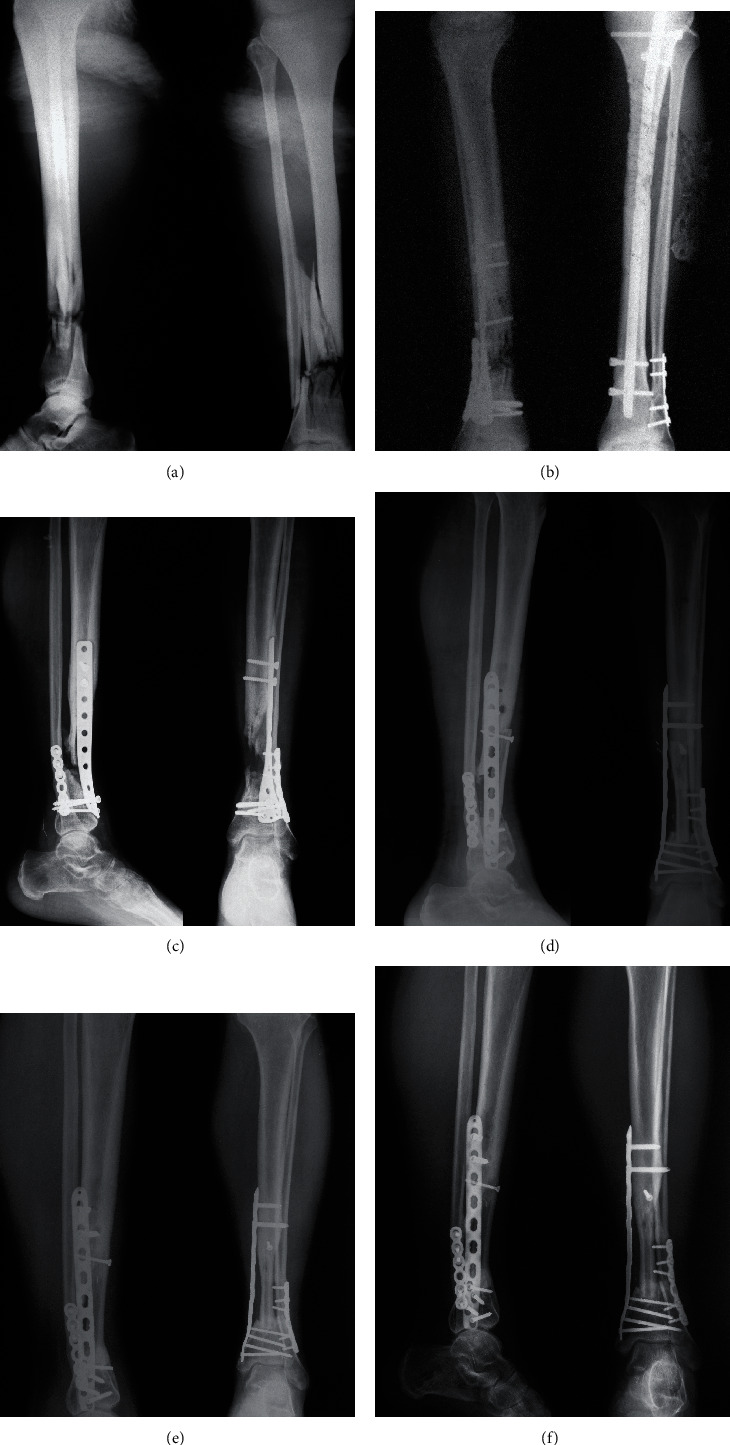
Case 8 in the septic group. (a) Radiograph showed initial left open tibiofibular fractures. (b) Radiograph of the left tibia and fibula 1 month after initial fixation. (c) Radiograph of the left tibia 15 months after initial fixation showed nonunion of the tibia and bone gap. (d) Radiograph of the left tibia 1 month after revision with FVFG and compression plating. A trough on the proximal tibia cortex was made as a docking site for the fibular graft. In this case, to prevent too deep insertion of the graft in the distal tibia and limb shortening, ATD was used to achieve controlled compression on the fibular graft. (e) Radiograph of the left tibia 10 months after revision. (f) Radiograph of the left tibia 12 months after revision showed healing of the fracture.

Compression was a critical step during plate osteosynthesis. We performed compression at the nonunion site in each case to minimize the bone gap and facilitate healing. All fibrous and scar tissue around the nonunion was removed, and trimming at the fracture ends was performed to increase bony contact before compression. Standard dynamic compression through the plate was applied when there was no segmental bone defect. In general, this was achieved by prebending the plate and applying one regular cortical screw eccentrically through a dynamic hole on each side of the fracture. In some cases, an articulated tension device (ATD, Synthes) might be used alone to achieve controlled compression (Figure 3) or with the dynamic compression technique to maximize compression between fragments (Figure 2). Occasionally, in cases with compromised bone quality due to osteoporosis, prolonged nonunion, or multiple surgical interventions, locking screws might be used to secure the plate to the fragment before compression with ATD (Figure 1). Locking screws may provide better purchase in these situations [12]. When there was a wedge defect, compression was also performed on the remaining cortex and cancellous bone grafting (ICBG) was applied if the defects were large [9]. In the condition of segmental defect, free vascularized fibular graft (FVFG) was used and a trough was created on the cortex of the femur or tibia as a docking site before inset of the fibular graft. Compression was achieved at both ends of the fibular graft to facilitate healing by using compression holes on the plate or ATD [13]. After compression, the fibular graft was fixed to the docking site by one cortical screw (Figure 3).

For all cases in this study, bone grafting methods are documented as iliac crest bone grafting (ICBG), free vascularized fibular graft (FVFG), and nongrafting. The use of demineralized bone matrix (DBM) or platelet-rich plasma (PRP) without ICBG or FVFG was also documented. The grafting method was highly individualized according to the bone defect, comminution, patient's background, and history of previous interventions. Generally, for nonunion with minor displacement and relatively intact cortices, compression between the fragments was achieved without bone grafting. ICBG was used for patients with wedge defects less than 4 cm and cases with prolonged nonunion time, history of multiple revision surgeries, and poor vascularity at the fracture ends to promote healing. Bioactive materials (DBM or PRP) may also be used as adjunctive methods. Free vascularized fibular grafting (FVFG) was used in cases with large or segmental bone defects (usually >4 cm) and patients who had failed from other grafting methods.

Patients were followed up in a monthly manner for the first 6 months postoperatively and then at a 2-month interval until complete healing was achieved. Range of motion exercises of the hip, knee, and ankle were started on the second postoperative day. Time to weight bearing was dependent on the healing process and was generally delayed to 2-3 months after surgery. Healing was defined by both the radiographic and clinical manifestations. The presence of bridging callus across the fracture in both AP and lateral views on X-rays was considered as radiographic union. Clinical union was defined as the absence of local tenderness at the nonunion site and full weight bearing without pain.

## 3. Results

A total of 54 patients were involved in this study. There were 14 females and 40 males with an average age of 39.65 years (range from 13 to 70). There were 46 aseptic and 8 septic nonunions. A total of 30 femoral and 24 tibial nonunions were included. The mechanism of injury consisted of 42 road traffic accidents, 3 falls, 6 fall from a height, and 3 crush injuries. Among all patients, there were 16 smokers (29.6%) and 6 patients (11.1%) with metabolic comorbidities. General information of each case in the aseptic and septic groups is listed in Supplementary Tables 1 and 2, respectively.

For aseptic nonunions, approximately 69.6% (32 of 46) cases were type B or C fractures according to AO/OTA classification. Four cases were open fractures. 28 of the 46 aseptic nonunions were located at either the proximal or distal third of the diaphysis. Fracture characteristics including AO/OTA classification, classification of open fractures, fracture locations, and primary fracture treatments for the aseptic group are summarized in Table 1.

In the aseptic group, there were 27 hypertrophic, 14 oligotrophic, and 5 atrophic nonunions according to the Weber and Cech classification. Eight cases had revision surgeries at other institutes before being enrolled in our hospital. In the aseptic group, 21 cases were initially fixed by nailing (with or without cable cerclage fixation) and 11 of them had fractures located at either the proximal or distal third of the diaphysis. This result indicates that the nonisthmus regions are easier to develop nonunion after nailing due to instability caused by either a wider canal or inappropriate nailing technique. There were 19 cases initially treated by plating, and traditional dynamic compression plate (DCP) with a principle of rigid fixation was applied in 16 of these 19 cases. These observations indicate that damage of blood supply during excessive dissection is likely the main reason for nonunions after plating. The remaining 6 cases in the aseptic group had either external fixation (5 cases) and/or nonoperative treatment with cast (1 case). Through radiological study, we found that in patients after nailing (21 cases), 16 cases (76.2%) had problems of inappropriate fixation, 2 with poor bone contact, and 3 with both. In patients after plating (19 cases), 13 cases (68.4%) had problems of inappropriate fixation, 2 with poor bone contact, and 4 with both. These results further suggest that surgery-related instability of fixation exists in the majority of our nonunion cases. Other fixation problems identified on the radiographs in the aseptic group included malalignment, screw pullout, and implant breakage and are listed in Table 2.

The bone grafting methods in the aseptic group are summarized in Table 3. In all 46 cases, 16 were treated without bone grafting. Three cases had only bone grafting substitutes (DBM, 2 cases; PRP, 1 case). Twenty-three of the 46 cases received ICBG with 2 of them using DBM at the same time. The remaining 4 cases were treated with FVFG. All 46 aseptic cases achieved healing uneventfully with no need for secondary surgery. The average union time was 8.28 months.

There were 4 open and 4 closed fractures in the group of infected nonunions. All the fractures were complex (AO/OTA classification type B or C). There were 7 atrophic and 1 hypertrophic nonunions. Only 2 cases were nondraining, and the remaining 6 cases were all with active draining infections. *Staphylococcus aureus* remains to be the most common organism identified (Supplementary Table 2). Two patients (Cases 3 and 4 in Supplementary Table 2) had preoperative malalignments. No implant breakage or screw pullout was observed in the septic group. Two-stage surgeries were performed for all infected nonunions. FVFG was applied in 5 cases while ICBG was used in the other 3 cases. All septic nonunions healed uneventfully. The average union time was 10.25 months.

All 54 patients returned to their preinjury activity level postoperatively. There was no complication reported at the bone graft harvest site. No recurrence of infection or any wound complication was found in our case series. We did not observe any malalignment, limb length discrepancy greater than one centimeter, or limited joint range of motion during the follow-up.

## 4. Discussion

Long bone diaphyseal nonunion of the lower extremities is one of the most common complications after fracture and is usually associated with a very low health-related quality of life [1]. The treatment is challenging and there is no ideal method, so far. Unlike exchange nailing, there is not as much literature looking at compression plating in the treatment of diaphyseal nonunions of the femur and the tibia [14].

The effectiveness of compression by nailing or plating in the nonunion treatment has been recognized several decades ago [15, 16]. But since then, due to a high complication rate of plating, nailing was preferred over compression plating in treating diaphyseal long bone nonunions of the lower limbs [3, 11, 15]. Although rarely reported, compression plating appears to show good results in the treatment of femur and tibia diaphyseal nonunions with the evolvement of modern plating techniques [10, 17]. Bellabarba et al. [17] reported successful treatment of 23 aseptic femoral nonunions after intramedullary nail fixation by compression plating with conventional DCPs. Ramoutar and colleagues [10] found a high union rate with compression plating in treatment of both upper and lower limb long bone nonunions, but their main focus was the advantages of decortication rather than the technique of compression plating. None of these studies included cases with segmental bone defects or severe infections. Despite the encouraging results, these reports have been published for over a decade. The efficacy of compression plating in treating these nonunions needs further investigation.

By focusing on the compression technique, our study provides the latest evidence on the effectiveness of modern plating technique in the treatment of femur and tibia diaphyseal nonunions. Compression is of fundamental importance in plate osteosynthesis. Biomechanical instability is an important factor leading to nonunion, and we also showed that fixation-related instability occurred in the majority of our cases. Adequate compression reduces the strain and improves the stability at the nonunion site [18]. Compression allows the bone itself to absorb axial compressive load, thus decreasing the strain on the plate and increasing the stability of the whole construct. Furthermore, better bone contact after compression also facilitates bone apposition and may create a favorable environment for bone grafts to heal. In this study, we also recommend applying compression using ATD. The degree of compression is controllable by using ATD, thus allowing the surgeons to perform compression in different situations such as cases with fibular grafts. ATD combined with dynamic compression through the plate provides maximum compression at the nonunion sites and is suitable for nonunions without significant bone loss.

To date, exchange nailing is still the method supported by the highest level of evidences in the treatment of diaphyseal nonunions of the tibia and the femur [19]. Advantages of exchange nailing include increased periosteal blood flow and new-bone formation after reaming as well as greater bending rigidity and strength by using a larger-diameter nail [6, 20]. However, some studies showed significant number of cases needed additional surgical procedures to achieve healing after exchange nailing [5, 21]. Failure of exchange nailing has specifically been noted in nonunions associated with extensive comminution, large segmental defects, and metaphyseal-diaphyseal junctional fractures [6]. The use of exchange nailing also has limitations. It has been reported that bone gap of more than 5 mm and an atrophic/oligotrophic pattern of nonunion were risk factors for failure of exchange nailing [21]. Moreover, exchanging with a nail of larger diameter cannot be done if the nail already inserted is of the largest diameter as marketed by the manufacturer [22]. This problem is even prominent in some underdeveloped regions where nails with proper sizes were not available.

Another treatment option is augmentative plating with nail in situ. The retained nail acts as a load-sharing device, neutralizing shear forces and maintaining alignment of the fracture [23]. Adding a plate provides additional stability when there is excessive motion at the nonunion site. Dynamic compression is also recommended for augmentative plating [24–26]. However, it is technically demanding to insert sufficient bicortical screws through the plate with a retained nail, especially in cases with midshaft fractures and large diameter nails [26]. Furthermore, it is difficult to correct angular deformity with a nail in situ [27]. Augmentation plating also has to be used with bone grafting in certain cases to promote healing, especially when local vascularity is poor [25, 26, 28]. Due to the low evidence level of the reports on augmentative plating [22, 29–31], a prospective controlled study is needed to further investigate the effectiveness and proper indications of this technique.

Our study provided an alternative option to treat tibia and femur diaphyseal nonunions by using compression plating with removal of previous implants. Implant removal, debridement, and plating facilitated correction of malreduction, interfragmentary compression, and bone grafting. Compared to nailing, compression plating provides a biomechanically superior tension band construct. Furthermore, the modern design of locking plates may also contribute to the success of nonunion revision surgeries. The limited-contact design of LCPs has lower infection rate [32] and less damage to the periosteal blood supply than traditional DCPs. Locking plates also have better angular and rotational stability than nailing, especially for fractures at the nonisthmus regions. The major drawbacks of compression plating are delayed weight bearing, more surgical dissection, and requirement of better soft tissue coverage.

Biological stimulation is also important for nonunion treatment. Compression plating facilitates different ways of bone grafting, such as cancellous bone grafting (ICBG) for wedge defects and structural grafting with FVFG for segmental defects shown in our case series. Judet decortication without bone grafting is also reported to be effective when used with compression plating in treating long bone nonunions [10]. Plating allows surgeons to choose a way of biological simulation freely according to aetiology and morphology of nonunion. Moreover, bone grafting with plates may be technically easier than with nailing. We and others [33, 34] have reported using fibular grafting in reconstructing bone defects in lower extremities. The current study further showed that compression plating combined with FVFG was an effective method to treat nonunions with large segmental defects. As previously reported [13], controlled compression was applied on the fibular graft and we believe that adequate compression may promote union and hypertrophy of vascularized fibular grafts (Figure 3).

Infection can result in nonunion due to direct action of bacteria and their products to the callus, osteolysis evoked by proinflammatory cytokines, delayed fracture repair, and compromised stability of the fixation [35]. Currently, the use of exchange nailing in the treatment of infected long bone nonunion is controversial [6]. However, bone transport using the Ilizarov method has been proven to be effective in treating infected nonunions [36]. We showed in this study that a two-stage strategy and reconstruction with compression plating and bone grafting were also effective in treating infected nonunions. Removal of implants and radical debridement with sequestrectomy are critical to eliminate infection in the first-stage surgery. Second-stage surgeries involved compression plating combined with ICBG or FVFG to reconstruct bone defect. Biomechanical stability plays critical roles not only in fracture healing but also in prevention and treatment of fracture-related infections [32]. Compression may contribute to the successful treatment of infected nonunions by increasing the stability of fixation. In our case series, no patient had infection recurrence and bone union was predictable. Compared to bone transport with ex-fix, plating does not have a long-lasting treatment period which may cause great patient discomfort. Cosmetic issues, second procedure to remove the frame, and several years to regain function after frame removal are the other disadvantages of the Ilizarov method. The drawback of our technique is that plating with FVFG is also technically demanding and requires a long time for the fibular graft to be hypertrophic to allow full loading.

The weaknesses of our study are the retrospective nature, absence of a control group, and small number of patients. Another limitation is that mixed types and locations of nonunions as well as various bone grafting methods were included and discussed together. Prospective case control studies are needed to further investigate this method and to better define its clinical indications.

## 5. Conclusion

In conclusion, our study showed successful treatment of both infected and noninfected diaphyseal nonunions of the tibia and the femur by compression plating with LCPs. Adequate compression by different techniques is important for stabilizing nonunion. Compression plating is also compatible with different bone grafting methods, and proper compression technique with bone grafting procures healing in cases with various bone defects. Our study provides a good alternative solution for the surgery of tibia and femur diaphyseal nonunions.

## Figures and Tables

**Table 1:** Summary of initial fracture characteristics (AO/OTA classification, Gustilo-Anderson classification, and fracture location) and primary fracture treatments (IMN, plate, ex-fix, and cast) for the aseptic group.

	Location	IMN	Plate	Ex-fix	Cast	Total
Total		21	19	5	1	46
*AO/OTA*						
A	Proximal or distal	5	2	0	0	7
	Middle	4	2	0	1	7
B	Proximal or distal	4	5	0	0	9
	Middle	5	1	1	0	7
C	Proximal or distal	2	7	3	0	12
	Middle	1	2	1	0	4
*Gustilo-Anderson*						
Close	Proximal or distal	10	13	3	0	26
	Middle	9	5	1	1	16
Open II	Proximal or distal	1	1	0	0	2
	Middle	1	0	0	0	1
Open IIIb	Proximal or distal	0	0	0	0	0
	Middle	0	0	1	0	1

**Table 2:** Number of cases with malalignment, screw pullout, and implant breakage before revision surgery in the aseptic group.

	Malalignment^∗^	Screw pullout	Implant breakage
Nail	5	1	1
Plate	8	7	8
Ex-fix	2	0	0

^∗^Angulation greater than 5 degrees on either AP or lateral X-ray was regarded as malalignment.

**Table 3:** Summary of grafting methods in the aseptic group according to fixation problems, no. of previous revision, Weber and Cech classification, and duration of nonunion (months).

	None	ICBG	FVFG	DBM/PRP	Total
Total	16	23	4	3	46
*Fixation problems*					
Inappropriate fixation	13	18	0	1	32
Poor bone contact	1	2	2	0	5
Both	2	3	2	2	9
*No. of previous revision*					
0	14	18	3	3	38
1-2	2	4	0	0	6
≥3	0	1	1	0	2
*Weber and Cech*					
Hypertrophy	13	14	0	0	27
Oligotrophy	3	7	1	3	14
Atrophy	0	2	3	0	5
*Duration of nonunion (months)*					
9-12	13	10	2	0	25
13-24	3	11	1	2	17
≥25	0	2	1	1	4

## Data Availability

Data are included in this published article and are available from the corresponding author.

## Supplementary Materials

Supplementary Materials Supplementary Table 1 and Supplementary Table 2 contain the general information of each case in the aseptic and septic groups, respectively.
