# Supplementary material for: Revision with Locking Compression Plate by Compression Technique for Diaphyseal Nonunions of the Femur and the Tibia: A Retrospective Study of 54 Cases
Source: Biomed Res Int. 2021 Jul 14;2021:9905067. doi: 10.1155/2021/9905067 (PMC8346318; doi:10.1155/2021/9905067)
Supplement: Supplementary Materials — Supplementary Table 1 and Supplementary Table 2 contain the general information of each case in the aseptic and septic groups, respectively. [file 9905067.f1.zip › supplementary table 1.pdf]

Supplementary Table 1. The general information of patients with aseptic nonunions.

Case	Age	Gender	Location	AO/OTA	GA	Previous treatment	Smoker	Comorbidites	Problems related to nonunion on imaging	No. previous revisions	Web & Cech	Duration of nonunion (months)	Bone grafting	Healing	Time to union (months)
1	29	F	Femur shaft, middle	32B	Close	IMN, wire	No	None	Inappropriate fixation	0	Hypertrophy	14	ICBG	Yes	6
2	49	M	Tibia shaft, middle	42B	Close	IMN	Yes	None	Inappropriate fixation, poor bone contact	0	Oligotrophy	13	PRP	Yes	8
3	35	M	Femur shaft, middle	32A	Close	IMN	Yes	None	Inappropriate fixation	0	Oligotrophy	15	ICBG	Yes	6
4	32	F	Femur shaft, middle	32B	Close	IMN	No	None	Inappropriate fixation	0	Hypertrophy	9	None	Yes	8
5	43	M	Femur shaft, middle	32A	Close	Plate (DCP)	No	None	Inappropriate fixation	0	Oligotrophy	15	None	Yes	7
6	21	M	Femur shaft, distal	32A	Close	Plate (DCP)	No	None	Inappropriate fixation	0	Hypertrophy	26	ICBG	Yes	6
7	35	F	Tibia shaft, middle	42B	Close	IMN	No	Metabolic Sydrome	Inappropriate fixation	0	Hypertrophy	12	ICBG	Yes	7
8	35	M	Femur shaft, distal	32A	Open Ⅱ	IMN	No	None	Inappropriate fixation	0	Oligotrophy	14	ICBG, DBM	Yes	7
9	33	M	Femur shaft, distal	32A	Close	IMN	No	None	Inappropriate fixation	0	Oligotrophy	23	ICBG	Yes	6
10	54	M	Tibia shaft, middle	42B	Open Ⅲb	Ex-fix	No	None	Inappropriate fixation	0	Atrophy	13	ICBG	Yes	9
11	54	F	Tibia shaft, distal	42B	Close	IMN, wire	No	None	Inappropriate fixation	0	Hypertrophy	14	None	Yes	8

12	31	M	Femur shaft, middle	32B	Close	Plate (DCP)	No	None	Inappropriate fixation	0	Hypertrophy	19	ICBG	Yes	6
13	61	M	Tibia shaft, middle	42A	Close	Plaster	Yes	None	Inappropriate fixation	0	Oligotrophy	12	ICBG, DBM	Yes	6
14	44	M	Tibia shaft, distal	42B	Close	Plate (LCP)	No	None	Inappropriate fixation	0	Hypertrophy	11	None	Yes	10
15	29	F	Tibia shaft, distal	42C	Close	Ex-fix	No	None	Inappropriate fixation, Poor bone contact (Bone defect)	1	Atrophy	11	ICBG	Yes	7
16	40	M	Tibia shaft, middle	42C	Close	Ex-fix, screw, wire	No	None	Inappropriate fixation, Poor bone contact (Bone defect)	3	Oligotrophy	36	FVFG	Yes	7
17	27	M	Femur shaft, proximal	32B	Close	IMN, wire	No	None	Inappropriate fixation	0	Hypertrophy	24	ICBG	Yes	6
18	53	F	Femur shaft, distal	32B	Close	IMN	No	Diabetes	Inappropriate fixation, poor bone contact	0	Oligotrophy	14	DBM	Yes	11
19	47	M	Tibia shaft, distal	42C	Close	Plate (LCP)	Yes	Diabetes	Inappropriate fixation	0	Hypertrophy	12	ICBG	Yes	7
20	24	M	Tibia shaft, middle	42A	Close	IMN	No	None	Inappropriate fixation	0	Oligotrophy	16	None	Yes	9
21	50	M	Tibia shaft, middle	42C	Close	Plate (DCP)	Yes	None	Poor bone contact （Bone defect）	0	Atrophy	13	FVFG	Yes	10
22	24	M	Tibia shaft, proximal	42C	Close	Ex-fix, wire	No	None	Inappropriate fixation	0	Hypertrophy	12	None	Yes	9
23	13	F	Femur shaft, distal	32B	Close	Plate (DCP)	No	None	Inappropriate fixation	0	Hypertrophy	11	None	Yes	6

24	45	M	Tibia shaft, distal	42B	Close	Plate (DCP)	Yes	None	Inappropriate fixation, poor bone contact （Bone defect）	0	Oligotrophy	13	ICBG	Yes	11
25	45	M	Femur shaft, proximal	32A	Close	IMN, wire	Yes	None	Inappropriate fixation	0	Hypertrophy	18	ICBG	Yes	7
26	39	M	Femur shaft, distal	32C	Close	Ex-fix	Yes	None	Poor bone contact （Bone defect）	0	Atrophy	12	FVFG	Yes	12
27	42	M	Femur shaft, distal	32C	Close	Plate (DCP)	No	Diabetes	Inappropriate fixation, poor bone contact	0	Hypertrophy	10	ICBG	Yes	9
28	55	M	Femur shaft, distal	32B	Close	Plate (DCP)	Yes	None	Inappropriate fixation	0	Hypertrophy	14	None	Yes	11
29	36	M	Tibia shaft, proximal	42C	Close	Plate (DCP)	No	None	Inappropriate fixation	0	Oligotrophy	9	ICBG	Yes	9
30	36	M	Tibia shaft, middle	42C	Open Ⅱ	IMN	No	None	Inappropriate fixation	1	Hypertrophy	12	None	Yes	8
31	21	M	Femur shaft, distal	32A	Close	IMN	No	None	Inappropriate fixation	0	Hypertrophy	9	None	Yes	6
32	31	M	Tibia shaft, middle	42A	Close	IMN	No	None	Poor bone contact	0	Oligotrophy	11	None	Yes	8
33	49	M	Femur shaft, proximal	32B	Close	Plate (DCP)	Yes	None	Inappropriate fixation	0	Hypertrophy	9	None	Yes	5
34	62	F	Femur shaft, distal	32C	Open Ⅱ	Plate (DCP), Graft	No	Diabetes	Inappropriate fixation	0	Oligotrophy	28	DBM	Yes	9

35	35	M	Femur shaft, middle	32C	Close	Plate (DCP)	No	None	Inappropriate fixation, poor bone contact	1	Hypertrophy	12	ICBG	Yes	8
36	36	F	Femur shaft, middle	32A	Close	IMN	No	None	Inappropriate fixation, poor bone contact	1	Hypertrophy	11	ICBG	Yes	7
37	49	M	Femur shaft, middle	32A	Close	Plate (DCP)	Yes	None	Inappropriate fixation	1	Hypertrophy	16	ICBG	Yes	10
38	31	F	Tibia shaft, proximal	42A	Close	Plate (DCP)	No	None	Inappropriate fixation	0	Hypertrophy	12	None	Yes	8
39	62	F	Femur shaft, distal	32A	Close	IMN	No	None	Inappropriate fixation	0	Hypertrophy	9	None	Yes	7
40	56	F	Femur shaft, distal	32C	Close	Plate (DCP)	No	None	Poor bone contact, （Bone defect）	0	Atrophy	12	FVFG	Yes	10
41	45	M	Femur shaft, distal	32C	Close	Plate (DCP)	No	None	Inappropriate fixation, poor bone contact	0	Hypertrophy	11	None	Yes	7
42	55	M	Femur shaft, middle	32B	Close	IMN	Yes	None	Inappropriate fixation	1	Hypertrophy	12	ICBG	Yes	6
43	35	M	Femur shaft, proximal	32B	Close	IMN, wire	No	None	Inappropriate fixation	0	Hypertrophy	12	None	Yes	9
44	49	F	Femur shaft, proximal	32C	Close	IMN, wire	No	None	Poor bone contact	4	Hypertrophy	36	ICBG	Yes	10
45	48	M	Femur shaft, distal	32C	Close	IMN	Yes	None	Inappropriate fixation	0	Oligotrophy	12	ICBG	Yes	15
46	70	M	Tibia shaft, proximal	42C	Close	Plate (LCP)	Yes	Diabetes	Inappropriate fixation	0	Hypertrophy	12	ICBG	Yes	17
