# Supplementary material for: Revision with Locking Compression Plate by Compression Technique for Diaphyseal Nonunions of the Femur and the Tibia: A Retrospective Study of 54 Cases
Source: Biomed Res Int. 2021 Jul 14;2021:9905067. doi: 10.1155/2021/9905067 (PMC8346318; doi:10.1155/2021/9905067)
Supplement: Supplementary Materials — Supplementary Table 1 and Supplementary Table 2 contain the general information of each case in the aseptic and septic groups, respectively. [file 9905067.f1.zip › supplementary table 2.pdf]

Supplementary Table 2. The general information of patients with septic nonunions.

Case	Age	Gender	Location	AO/OTA	GA	Previous treatment	Smoker	Comorbiditeis	Problems	No. previous revisions	Web & Cech	Duration of nonunion (month)	Organisms	Draining	Bone grafts	Healing	Time to union (month)
1	44	M	Tibia shaft, distal	42C	Close	Plate	Yes	None	Inappropriate fixation	2	Atrophy	16	Staphylococcus aureus	Yes	ICBG	Yes	12
2	20	M	Tibia shaft, middle	42C	Open Ⅱ	Plate	No	None	Poor bone contact	2	Atrophy	12	Staphylococcus aureus	Yes	ICBG	Yes	10
3	41	F	Femur shaft, distal	32B	Close	Plate & IMN	No	None	Inappropriate fixation, poor bone contact	2	Hypertrophy	27	Klebsiella	Yes	FVFG	Yes	10
4	32	M	Tibia shaft, middle	42B	Open Ⅲa	IMN	No	None	Inappropriate fixation	2	Atrophy	14	Staphylococcus aureus	Yes	ICBG	Yes	7
5	21	M	Tibia shaft, middle	42C	Open Ⅲb	Ex-fix	No	None	Poor bone contact	2	Atrophy	10	Staphylococcus aureus	Yes	FVFG	Yes	8
6	15	M	Tibia shaft, middle	42C	Close	Ex-fix	No	None	Poor bone contact	6	Atrophy	21	Enterococcus faecalis	Yes	FVFG	Yes	12
7	31	M	Femur shaft, middle	32B	Close	IMN	No	None	Poor bone contact	2	Atrophy	18	Staphylococcus aureus	No	FVFG	Yes	11
8	42	M	Tibia shaft, distal	42C	Open Ⅱ	Plate	Yes	None	Inappropriate fixation, poor bone contact	2	Atrophy	12	Staphylococcus aureus	No	FVFG	Yes	12
